# Knowledge, attitude, and use of electronic cigarettes among Cairo University medical students

**DOI:** 10.1186/s42506-024-00177-5

**Published:** 2024-11-26

**Authors:** Ola A. Mostafa, Mahmoud A. Taha

**Affiliations:** 1 Public Health and Community Medicine, Cairo University, Cairo, Egypt; 2 Medical School Graduate, Cairo University, Cairo, Egypt

**Keywords:** Awareness, Electronic cigarette, Medical students

## Abstract

**Background:**

Electronic cigarette use has increased exponentially in Egypt and all over the world. However, there is insufficient information about their use among Cairo University medical students. This study aimed to assess e-cigarette knowledge, attitude, and use among Cairo University medical students.

**Methods:**

A cross-sectional study of 300 consenting Cairo University medical students in the period of 1st November 2023 to 15th December 2023 was conducted using a self-administered questionnaire. A multistage sampling technique was used to randomly select 300 students: 150 students from the third year and 150 from the fifth year of the Cairo University Medical School.

**Results:**

The mean age was 22.12 ± 1.87 years and 69.3% were males and 30.7% were females. Most of them (88.3%) had heard about e-cigarettes, with higher knowledge for students in their clinical years. The main sources of information were the media and friends (41.8% and 37.5%). By type, 14%, 12.7%, and 7.3% were cigarette, shisha, and e-cigarette smokers. Smoking prevalence was higher among males and students in their clinical years (12% and 4.7% in clinical and preclinical years respectively, *p* = 0.02).

Among all, 39.3%, 10.2%, and 3.3% of the participants’ friends, siblings, and parents smoked e-cigarettes respectively; compared to 16.7%, 17.9%, and 33% among the subset of participants who were e-cigarette smokers. A significantly lower percentage of e-cigarette smokers believed that e-cigarettes are addictive or cause respiratory problems, and a higher percentage thought that e-cigarettes are less harmful, help in smoking cessation, and that their nicotine content is lower than traditional cigarettes. Predictors of e-cigarette smoking were being in clinical years, cigarette and shisha smoking, and having friends who are e-cigarette smokers.

**Conclusion:**

The study revealed several students’ misconceptions and a wide variation in their attitudes about the harmful and addictive effects of e-cigarettes. This underscores the urgent need for the development of a medical school curriculum to provide accurate information about e-cigarettes to address the problem of its growing use in Egypt.

## Introduction

Despite the controversy about the health risks related to electronic cigarette use, the increasing popularity of e-smoking is considered a potential public health threat [1]. In the USA, e-cigarettes were found to be the most commonly used tobacco product among middle and high school students [2]. A study revealed that e-cigarette prevalence in the USA in 2015 was 27% [3]. In Great Britain, a study (2014) revealed that 20% of the participants were e-cigarette users and that the majority of smokers were aware of e-cigarettes[4]. In Ireland, 12% of all respondents had tried e-cigarettes[5]. In Canada, 16.1% of the participants reported trying an e-cigarette [6]. A study in Egypt (2016) showed that 57.5% of the participants had heard about e-cigarettes. In Saudi Arabia, 68.9% of the smoker participants used e-cigarettes [7].

E-cigarettes are battery-operated devices that can deliver vaporized nicotine that is inhaled by the user. They are composed of a liquid cartridge, a pipe stub, an energy source, and an electronic circuit that evaporates the liquid in the cartridge. This liquid consists of glycerol, a variety of flavors, and different concentrations of nicotine ranging between 0 and 26 mg [8]. Although tobacco is not burnt in e-cigarettes, it includes some carcinogenic and toxic materials such as nitrosamine and diethylene glycol found in the e-liquids[9, 10].

The increasing use of e-cigarettes is due to their similarity to conventional tobacco cigarettes [11]. In addition, the variety of flavors attracts adolescents and young adults. Moreover, the perception that e-cigarettes are less harmful than conventional smoking, and the lack of regulations for their indoor use further increase their popularity [9].

E-cigarettes were initially developed as an alternative tool to help in smoking cessation. However, research results have indicated that the use of e-cigarettes by adolescents was found to increase their susceptibility to using traditional cigarettes [12].

To date, the long-term health consequences of e-smoking remain unstudied due to the relatively short duration since e-cigarettes have been available [1]. Previous research indicated that frequent and even short-term exposure to vapors of e-cigarettes induces an immune response similar to that induced by traditional cigarettes. Such a response could further trigger a systematic and pulmonary immune response [13]. Various amounts of carcinogenic compounds, such as diethylene glycol, lead, chromium, nickel, and tobacco-specific N-nitrosamines, have been detected in the mist and solution of e-cigarette cartridges [14, 15].

Pod mods are a new generation of e-cigarettes that captured 50% of the e-cigarette market, especially among adolescents and young adults [16]. They are small rechargeable devices that aerosolize liquid solutions containing nicotine, flavoring, and other contents encapsulated in cartridges. Juul which is a type of Pod mod, is small-sized and resembles a computer USB, which makes it easy for adolescents in schools to conceal it from parents and authorities. Furthermore; Juuls contain high levels of nicotine equivalent to approximately 20 combustible cigarettes [17]. The World Health Organization (WHO) recommends restrictions on e-cigarette advertisements in media as well as their sales to minors [18]. On April 18, 2018, six public health organizations urged the Food and Drug Administration (FDA)—which is the federal agency charged with regulating e-cigarettes—to take action to prevent “Juul-ing” by young people. They urged the FDA to prohibit internet sales of Juul until stronger regulations can be enforced [19].

Currently, there is much debate about electronic cigarettes in both public health and academic communities. The WHO reported many concerns about e-cigarette use; including health risks, their effectiveness as smoking cessation aids, their potential for causing Nicotine addiction among smokers, and their ability to attract young non-smokers to initiate nicotine use [20].

With the increasing prevalence of e-cigarette use, healthcare providers should be prepared to provide accurate evidence-based advice to their patients regarding this emerging issue [21]. E-cigarette knowledge, attitude, and practice (KAP) is of particular importance among medical students due to their greater contact with the youth compared to practicing physicians. Additionally, medical students are the future healthcare providers who will be in charge of smoking cessation efforts [20].

An understanding of the deficits in knowledge and misperceptions among medical students about e-cigarettes will serve as a guide for the healthcare authorities to provide the necessary medical education. The purpose of this study was to investigate the knowledge, perceptions, and practice of e-cigarettes among medical students at Cairo University Medical School.

## Methods

### Study design

This study was a cross-sectional analytical study conducted at Cairo University Medical School from November 1st, 2023 till December 15th, 2023.

The study included 300 consenting Cairo University Medical School students; 150 from the third year and 150 from the final year of Medical School.

The choice was based on the idea of comparing awareness, attitude, and use of e-cigarettes among students who had completed their academic years of study versus those who had also completed their clinical years of study. Participation was voluntary and the aim of the study was explained to the students. Informed consent was obtained from all participants.

### Sample size

The sample size was calculated using the Med Calc program with a presumed 6.2% e-cigarette use among medical students, similar to a previous study performed in Pakistan [22]. Using a 5% precision, 80% power, and an alpha of 0.05, the minimal required sample size was calculated to be 270. The final sample size used was 300 students including 150 students from the preclinical years and 150 from the clinical years.

### Sampling technique

A multistage sampling technique was used to randomly select 300 students: 150 students from the third year, and 150 students from the fifth year of the Cairo University medical school. The multistage sampling was performed as follows:

1. Students were stratified into 2 strata by grade (third and fifth)
2. Students were then divided into clusters according to the assigned rotation.

The total number of third-year students was 1339, who were divided into four clusters (334 students each) according to the assigned rotation, then one cluster was randomly selected from the 4 clusters.

The total number of fifth-year students was 1275; who were divided into thirteen clusters (98 students each) according to the assigned rotation. Two clusters were then randomly selected from the 13 clusters.

3) A simple random sample was then chosen from each of the selected clusters: 150 students were chosen from the third-year students’ cluster and a similar 150 students were chosen from the 2 clusters for the final-year students.

### Study tool

A self-administered pre-coded questionnaire was used to explore knowledge, perception, and use of e-cigarettes among medical students.

It included baseline characteristics such as age, sex, academic year, residence during the academic year, smoking status (frequency, duration, and type of smoking), as well as the smoking status of parents, siblings, and friends. Knowledge questions included inquiries about e-cigarette knowledge and the source of such knowledge, questions about the harmful effects of e-cigarettes compared to traditional cigarettes, the association between e-cigarettes and addiction/dependency, the nicotine content of e-cigarettes, and PODs. Attitude questions included inquiries about whether e-cigarettes aid in smoking cessation and if they would recommend it to any of their friends, in addition to their perception of whether e-cigarettes are less harmful than traditional cigarettes.

A pilot test was performed on 20 students to check the clarity of the questions. The required modifications were applied, and those results were not included in the study sample.

### Statistical analysis

Data were analyzed using the IBM SPSS Statistics 21 software program. The data was summarized using mean and standard deviation for quantitative normally distributed variables, while median and interquartile range (IQR) were used for quantitative variables, which were not normally distributed. Numbers and percentages were used for qualitative values. A chi-squared (χ^2^) test was used to compare qualitative variables. Logistic regression analysis was done to test for predictors of e-cigarette smoking. *p*-values less than or equal to 0.05 were considered statistically significant.

## Results

The mean age of the study participants was 22.12 ± 1.87 years, with students in the preclinical years averaging 20.33 ± 0.88 and those in the clinical years averaging 23.74 ± 0.66. About two-thirds (69.3%) were males and one-third (30.7%) were females. The majority of students (87%) reported being aware of e-cigarettes, with a higher percentage of awareness among students in the clinical years compared to those in the preclinical years (89.3% versus 84.7% respectively); with no statistical significance (*p* = 0.21). Additionally, a statistically significantly higher percentage of students in the clinical years were aware of PODs compared to students in the preclinical years (45.3% vs 17.3% respectively, *p* < 0.001). The main sources of awareness of e-cigarettes and PODs were the media followed by friends (41.8% and 37.5% for e-cigarettes and 54.8% and 30.1% for PODs). There were statistically significant differences in the source of knowledge between students in the preclinical and clinical years, with *p* < 0.001.

Friends constituted the highest percentage of smokers (both traditional cigarettes and e-cigarettes) among students (43.3% and 39.3%). Moreover, 28% and 3.3% of the participants’ parents smoked traditional and electronic cigarettes respectively while 17.5% and 10.2% of the participants’ siblings smoked traditional and e-cigarettes respectively (Table 1).

**Table 1:** Baseline characteristics and knowledge about e-cigarettes among medical students in preclinical and clinical years at Cairo University Medical School in the academic year 2023

Baseline characteristics and knowledge about E-cigarettes	Grade				*p* value	Total	
	Third year		Fifth year				
	No. = 150	%	No. = 150	%		No. = 300	%
Sex							
Male	92	61.3	116	77.3	0.003	208	69.3
Female	58	38.7	34	22.7		92	30.7
Age (years)	20.33 ± 0.88		23.74 ± 0.66		< 0.001	22.12 ± 1.87	
Residence							
Alone	20	13.3	12	8.0	0.47	32	10.7
With friend	20	13.3	21	14.0		41	13.7
With family	106	70.7	111	74.0		217	72.3
Other	4	2.7	6	4.0		10	3.3
Heard about e-cigarettes							
*Yes*	127	84.7	134	89.3	0.21	261	87.0
Knowledge source (*n* = 261)							
Friends	49	38.6	49	36.6	< 0.001	98	37.5
Media	61	48.0	48	35.8		109	41.8
Other	6	4.7	1	0.7		7	2.7
Friends and media	11	8.7	36	26.9		47	18.0
Heard about PODs							
*Yes*	26	17.3	68	45.3	< 0.001	94	31.3
Knowledge source							
Friends	9	36.0	19	27.9	0.006	28	30.1
Media	10	40.0	41	60.3		51	54.8
Other	5	20.0	1	1.5		6	6.5
Friends and media	1	4.0	7	10.3		8	8.6
Does any of the following persons around you smoke traditional Cigarettes?^##^							
Parents	25	19.7	52	35.1	0.004	77	28.0
Siblings	20	15.7	28	18.9	0.49	48	17.5
Friends	72	56.7	47	31.8	< 0.001	119	43.3
Does any of the following around you smoke *e-cigarettes?*^##^							
Parents	6	4.7	3	2.0	0.31	9	3.3
Siblings	13	10.2	15	10.1	0.98	28	10.2
Friends	52	40.9	56	37.8	0.6	108	39.3

^##^Percent of smoking was calculated for each answer separately

More than 80% (83.3%) of the students were non-smokers, more than one-tenth (12.7%) were current smokers, and a minority (4%) were ex-smokers. Cigarette smokers constituted 14% of the students, among them 47.6% smoked cigarettes daily. There was a statistically significant difference in smoking frequency between clinical and preclinical years. Shisha smokers constituted 12.7% of the students, among them 7.9% use shisha daily. E-cigarette smokers constituted 7.3% of the students, among them 13.6% used e-cigarettes daily. A statistically significant higher percentage of students in the clinical years used e-cigarettes compared to students in the preclinical years (11.3% vs3.3% respectively, *p* = 0.008). POD smokers constituted 4.3% of students, among them 8.3% used PODs daily. Students who used e-cigarettes and PODs constituted 8.3%. A statistically significantly higher percentage of students in the clinical years used both e-cigarettes and PODs compared to students in the preclinical years (12% and 4.7% respectively, *p* = 0.02), (Table 2).

**Table 2:** Smoking status of medical students in the preclinical and clinical years at Cairo University Medical School in the academic year 2023

Smoking status of medical students	Grade				* P* value	Total	
	Third year		Fifth year				
	No.	%	No.	%		No.	%
Smoking status							
Current smoker	15	10.0	23	15.3	0.16	38	12.7
No	131	87.3	119	79.3		250	83.3
Ex-smoker	4	2.7	8	5.3		12	4.0
Cigarette							
Yes	18	12.0	24	16.0	0.32	42	14.0
No	132	88.0	126	84.0		258	86.0
Cigarette frequency							
Daily	5	27.8	15	62.5	0.04*	20	47.6
Sometimes	4	22.2	5	20.8		9	21.4
Rarely	9	50.0	4	16.7		13	31.0
Shisha							
Yes	19	12.7	19	12.7	1	38	12.7
No	131	87.3	131	87.3		262	87.3
Shisha frequency							
Daily	2	10.5	1	5.3	0.42	3	7.9
Sometimes	11	57.9	8	42.1		19	50.0
Rarely	6	31.6	10	52.6		16	42.1
e-cigarettes							
*Yes*	5	3.3	17	11.3	0.008*	22	7.3
*No*	145	96.7	133	88.7		278	92.7
e-cigarettes frequency							
Daily	1	20.0	2	11.8	0.10	3	13.6
Sometimes	0	0.0	9	52.9		9	40.9
Rarely	4	80.0	6	35.3		10	45.5
POD							
Yes	4	2.7	9	6.0	0.16	13	4.3
No	146	97.3	141	94.0		287	95.7
POD frequency							
Daily	0	0.0	1	11.1	0.8	1	8.3
Sometimes	1	33.3	2	22.2		3	25.0
Rarely	2	66.7	6	66.7		8	66.7
e-cigarettes and PODs							
Yes	7	4.7	18	12.0	0.02*	25	8.3
No	143	95.3	132	88		275	91.7

^*^p < 0.05

The median (Q1:Q3) duration for cigarette smoking and shisha among smokers was 3(2:5) years, while the median (Q1:Q3) duration for e-cigarette smoking and POD was 1(1:2) years and 1(1:1) year respectively (Table 3).

**Table 3:** Median smoking duration among medical students in preclinical and clinical years at Cairo University Medical School in the academic year 2023

Smoking type	3rd year median (Q1:Q3)	5th year median (Q1:Q3)	Total median (Q1:Q3)
Cigarettes	2(2:6)	3(3:5)	3(2:5)
Shisha	3(2:4.5)	3(3:5)	3(2:5)
E-cigarettes	1(0.5:2)	1(1:2)	1(1:2)
POD	1	1(1:1)	1(1:1)

A statistically significantly higher percentage of 5th-year medical students compared to those in the 3rd year perceived e-cigarettes as causing respiratory problems; reported that e-cigarettes are not harmful at all, and agreed that e-cigarettes help in smoking cessation.

On the other hand, a statistically significantly higher percentage of 3rd year medical students compared to those in the 5th year reported that e-cigarettes cause addiction; and are as harmful as traditional cigarettes.

A high percentage (70.6%) of the students reported that they do not know if the harmful effects of PODs differ from those of traditional cigarettes, with a statistically significant difference in both groups (Table 4).

**Table 4:** Perceived health problems caused by e-cigarettes among medical students in clinical and preclinical years at Cairo University Medical School in the academic year 2023

Perceived health problems caused by e-cigarettes	Grade				* P* value	Total	
	3rd year		5th year				
	No.	%	No.	%		No.	%
Respiratory problems							
No	19	13.2	9	6.0	0.04*	28	9.6
Don’t know	47	32.6	41	27.5		88	30.0
Yes	78	54.2	99	66.5		177	60.4
Addiction							
No	20	13.9	63	42.3	< 0.001**	83	28.3
Don’t know	61	42.4	46	30.9		107	36.5
Yes	63	43.7	40	26.8		103	35.2
e-cigarettes are less harmful than traditional cigarettes							
No	25	17.5	18	12.1	0.07	43	14.7
Don’t know	46	32.2	36	24.2		82	28.1
Yes	72	50.3	95	63.8		167	57.2
e-cigarettes are more harmful than traditional cigarettes							
No	77	53.8	93	62.8	0.14	170	58.4
Don’t know	52	36.4	38	25.7		90	30.9
Yes	14	9.8	17	11.5		31	10.7
e-cigarettes are as harmful as traditional cigarettes							
No	51	35.9	102	68.5	< 0.001**	153	52.6
Don’t know	68	47.9	42	28.2		110	37.8
Yes	23	16.2	5	3.4		28	9.6
e-cigarettes are not harmful at all							
No	88	61.5	101	67.8	0.03*	189	64.7
Don’t know	45	31.5	29	19.5		74	25.3
Yes	10	7.0	19	12.8		29	9.9
e-cigarettes are harmful to pregnant women							
No	6	4.2	5	3.4	0.2	11	3.8
Don’t know	53	37.3	42	28.2		95	32.6
Yes	83	58.5	102	68.5		185	63.6
E-cigarettes help in smoking cessation							
No	14	9.9	17	11.4	< 0.001**	31	10.7
Don’t know	74	52.1	38	25.5		112	38.5
Yes	54	38.0	94	63.1		148	50.9
Nicotine content of e-cigarettes compared to traditional cigarettes is							
Less	12	8.5	50	33.6	< 0.001**	62	21.4
Equal	7	5.0	5	3.4		12	4.1
More	5	3.5	10	6.7		15	5.2
Don’t know	117	83.0	84	56.4		201	69.3
The harmful effects of PODs compared to traditional cigarettes is							
Less	15	10.7	44	29.5	< 0.001**	59	20.4
Equal	4	2.9	7	4.7		11	3.8
More	6	4.3	8	5.4		14	4.8
Not Harmful	1	0.7	0	0.0		1	0.3
Don’t Know	114	81.4	90	60.4		204	70.6
I would advise smokers to use e-cigarettes	58	45.3	80	54.1	0.14	138	50.0
I would advise non-smokers to use e-cigarettes	12	10.3	27	18.4	0.07	39	14.8

^*^p < 0.05, **p < 0.001

Comparing perceived health problems of e-cigarettes among smokers, non-smokers, and ex-smokers (Table 5), a statistically significant lower percent of current smokers and ex-smokers reported that e-cigarettes cause respiratory problems compared to non-smokers (48.6%,50%, and 62.3% respectively, *p* < 0.001). Furthermore, a statistically significantly lower percentage of current smokers reported that e-cigarettes are addictive compared to ex-smokers and non-smokers (24.3%, 41.7, and 36.5%, *p* = 0.002).

**Table 5:** E-cigarette awareness among active smokers, ex-smokers, and non-smokers among medical students at Cairo University Medical School in the academic year 2023

E-cigarette awareness	Smoking status						* P* value
	Non-smoker		Ex-smoker		Current smoker		
	No.	%	No.	%	No.	%	
Respiratory problems							
No	18	7.4	5	41.7	5	13.5	0.001**
Don’t know	73	29.9	1	8.3	14	37.8	
Yes	153	62.7	6	50.0	18	48.6	
Addiction							
No	58	23.8	6	50.0	19	51.4	0.002*
Don’t know	97	39.8	1	8.3	9	24.3	
Yes	89	36.5	5	41.7	9	24.3	
The harmful effects of e-cigarettes are less than traditional cigarettes							
No	35	14.4	3	25.0	5	13.5	0.078
Don’t know	76	31.3	1	8.3	5	13.5	
Yes	132	54.3	8	66.7	27	73.0	
The harmful effects of e-cigarettes are more than traditional cigarettes							
No	140	57.6	6	50.0	24	66.7	0.37
Don’t know	79	32.5	3	25.0	8	22.2	
Yes	24	9.9	3	25.0	4	11.1	
The harmful effects of e-cigarettes are equal to that of traditional cigarettes							
No	118	48.8	10	83.3	25	67.6	0.029*
Don’t know	101	41.7	1	8.3	8	21.6	
Yes	23	9.5	1	8.3	4	10.8	
e-cigarettes are not harmful at all							
No	151	62.1	10	83.3	28	75.7	0.306
Don’t know	67	27.6	1	8.3	6	16.2	
Yes	25	10.3	1	8.3	3	8.1	
e-cigarettes are harmful to pregnant women							
No	10	4.1	1	8.3	0	0.0	0.645
Don’t know	80	33.1	3	25.0	12	32.4	
Yes	152	62.8	8	66.7	25	67.6	
e-cigarettes help in smoking cessation							
No	25	10.3	0	0.0	6	16.7	< 0.001**
Don’t know	108	44.4	2	16.7	2	5.6	
Yes	110	45.3	10	83.3	28	77.8	
Nicotine content of e-cigarettes compared to traditional cigarettes is							
Less	44	18.3	5	41.7	13	35.1	< 0.001**
Equal	7	2.9%	3	25.0	2	5.4	
More	11	4.6%	0	0.0	4	10.8	
Don’t know	179	74.3	4	33.3	18	48.6	
The harmful effects of PODs compared to traditional cigarettes are							
Less	44	18.3	5	41.7	10	27.0	0.001**
Equal	8	3.3	3	25.0	0	0.0	
More	10	4.2	0	0.0	4	10.8	
Not	1	0.4	0	0.0	0	0.0	
Don’t know	177	73.8	4	33.3	23	62.2	
I would advise smokers to use e-cigarettes							
Yes	109	48.0	8	66.7	21	56.8	0.306
I would advise non-smokers to use e-cigarettes							
Yes	35	16.1	0	0.0	4	11.4	0.258

^*^p < 0.05, **p < 0.001

A higher percentage of current smokers and ex-smokers reported that e-cigarettes are less harmful than traditional cigarettes compared to non-smokers (73%, 66.7%, and 45.3% respectively). However, this difference was not statistically significant. Moreover, it was noticed that a higher percentage of ex-smokers reported that e-cigarettes are more harmful than traditional cigarettes compared to current smokers and non-smokers (25%, 22.2%, and 9.9% respectively). However, these differences were not statistically significant.

The results revealed that there was a statistically significant difference between smokers, ex-smokers, and non-smokers in their awareness of whether e-cigarettes are as harmful as traditional cigarettes; *p* = 0.029. A statistically significant higher percentage of non-smokers reported that e-cigarettes cause respiratory problems; compared to current smokers and ex-smokers, *p* < 0.001.

A higher percentage of smokers and ex-smokers disagreed that that e-cigarettes are not harmful at all compared to non-smokers, however; these differences were not statistically significant.

A statistically significant higher percentage of ex-smokers and current smokers mentioned that e-cigarettes help in smoking cessation (83.3%,77.8% compared to 45.3% in non-smokers (*p* < 0.001).

A statistically significant higher percentage of ex-smokers and current smokers mentioned that the nicotine content of e-cigarettes is lower than traditional cigarettes compared to non-smokers (41.7%, 35.1%, and 18.3% respectively, *p* < 0.001).

A higher percentage of ex-smokers and smokers reported that they would advise smokers to use e-cigarettes (66.7% and 56.8%) compared to non-smokers (48%). However, these differences were not statistically significant (Table 5).

A higher percentage of males used e-cigarettes than females ((9.1% vs 6.5%). A higher percentage of 5th-year students used e-cigarettes than 3rd-year students (12% versus 4.7% with no significant difference). About half of the students who smoked e-cigarettes used shisha and traditional cigarettes as well. Among e-cigarette users, about one-third (33.3%) of their parents used e-cigarettes, and almost one-fifth (18.2%) smoked traditional cigarettes. Almost one-fifth of their siblings smoked e-cigarettes and traditional cigarettes (17.9% and 18.8% respectively). Their friends smoked e-cigarettes and traditional cigarettes at rates of 16.7% and 13.4% respectively (Table 6).

**Table 6:** Comparison of baseline characteristics and knowledge about e-cigarettes among e-cigarette users and non-users among medical students at Cairo University Medical School in the academic year 2023

Baseline characteristics and knowledge about e-cigarettes	Use of e-cigarettes and PODs				* P* value
	Yes		No		
	No.	%	No.	%	
Sex					
Male	19	9.1	189	90.9	0.5
Female	6	6.5	86	93.5	
Age	22.79 ± 1.93		22.05 ± 1.86		
Grade					
3rd year	7	4.7	143	95.3	0.02*
5th year	18	12.0	132	88.0	
Residence					
Alone	6	18.8	26	81.3	0.10
With friend	2	4.9	39	95.1	
With family	17	7.8	200	92.2	
Others	0	0.0	10	100.0	
Smoking status					
Current smoker	17	44.7	21	55.3	< 0.001**
No	6	2.4	244	97.6	
Ex-smoker	2	16.7	10	83.3	
Cigarette					
Yes	21	50.0	21	50.0	< 0.001**
No	4	1.6	254	98.4	
Shisha					
Yes	20	52.6	18	47.4	< 0.001**
No	5	1.9	257	98.1	
Does any of your parents smoke traditional cigarettes?					
Yes	14	18.2	63	81.8	0.001**
No	11	5.6	187	94.4	
Does any of your siblings smoke traditional cigarettes?					
Yes	9	18.8	39	81.3	0.02*
No	16	7.0	211	93.0	
Does any of your friends smoke traditional cigarettes?					
Yes	16	13.4	103	86.6	0.028*
No	9	5.8	147	94.2	
Does any of your parents use e-cigarettes?					
Yes	3	33.3	6	66.7	0.04*
No	22	8.3	244	91.7	
Does any of your siblings use e-cigarettes?					
Yes	5	17.9	23	82.1	0.15
No	20	8.1	227	91.9	
Does any of your friends use e-cigarettes?					
Yes	18	16.7	90	83.3	< 0.0001**
No	7	4.2	160	95.8	
Would you advise smokers to use e-cigarettes?					
Yes	15	10.9	123	89.1	0.29
No	10	7.2	128	92.8	
Would you advise non-smokers to use e-cigarettes?					
Yes	1	2.6	38	97.4	0.22
No	23	10.2	202	89.8	

^*^p < 0.05, **p < 0.001

Among e-cigarette users, a lower percentage was aware that e-cigarettes cause respiratory problems than among non-users (48% vs 61.6% respectively). Regarding the addictive effects of e-cigarettes, a better awareness was demonstrated by e-cigarette non-users; where a lower percentage of non-users reported that e-cigarettes do not cause addiction than e-cigarette users (26.9% vs 44% respectively). Additionally, a higher percentage of e-cigarette users reported that the harmful effects of e-cigarettes are less than traditional cigarettes (72%) in contrast to 55.8% among non-users. Furthermore, a statistically significantly higher percentage of POD users compared to non-users reported that the harmful effects of e-cigarettes and PODs are less than traditional cigarettes (48% versus 17.8% respectively, *p* = 0.011). A higher percentage of e-cigarette users reported that e-cigarettes are not harmful to pregnant women compared to non-users ((8% vs 3.4%) (Table 7).

**Table 7:** Responses to questions about e-cigarette awareness among users and non-users of e-cigarettes (and PODs) Among Medical Students at Cairo University Medical School in the academic year 2023

Questions about e-cigarette awareness	Use of e-cigarettes and POD				* P* value
	Yes		No		
	No.	%	No.	%	
Respiratory problems					
No	4	16.0	24	9.0	0.332
Don’t know	9	36.0	79	29.5	
Yes	12	48.0	165	61.6	
Addiction					
No	11	44.0	72	26.9	0.058
Don’t know	4	16.0	103	38.4	
Yes	10	40.0	93	34.7	
The harmful effects of e-cigarettes are less than traditional cigarettes					
No	1	4.0	42	15.7	0.188
Don’t know	6	24.0	76	28.5	
Yes	18	72.0	149	55.8	
The harmful effects of e-cigarettes are more than traditional cigarettes					
No	16	64.0	154	57.9	0.735
Don’t know	6	24.0	84	31.6	
Yes	3	12.0	28	10.5	
The harmful effects of e-cigarettes are equal to that of traditional cigarettes					
No	17	68.0	136	51.1	0.158
Don’t know	5	20.0	105	39.5	
Yes	3	12.0	25	9.4	
e-cigarettes are not harmful at all					
No	20	80.0	169	63.3	0.222
Don’t know	3	12.0	71	26.6	
Yes	2	8.0	27	10.1	
e-cigarettes are harmful to pregnant women					
No	2	8.0	9	3.4	0.510
Don’t know	8	32.0	87	32.7	
Yes	15	60.0	170	63.9	
e-cigarettes help in smoking cessation					
No	4	16.0	27	10.2	0.129
Don’t know	5	20.0	107	40.2	
Yes	16	64.0	132	49.6	
Nicotine content of e-cigarettes compared to traditional cigarettes is					
Less	12	48.0	50	18.9	< 0.001**
Equal	4	16.0	8	3.0	
More	1	4.0	14	5.3	
Don’t know	8	32.0	193	72.8	
The harmful effects of PODs compared to traditional cigarettes are					
Less	12	48.0	47	17.8	0.011*
Equal	1	4.0	10	3.8	
More	1	4.0	13	4.9	
Not	0	0.0	1	0.4	
Don’t know	11	44.0	193	73.1	

^*^p < 0.05, **p < 0.001

Logistic regression analysis was done to test for predictors of e-cigarette smoking among the studied group. Academic year, sex, cigarette smoking, shisha smoking, parents’ cigarette smoking, and friends’ e-cigarette smoking were entered into the regression model. Only sixth-year students compared to third-year students (*p* = 0.026, odds ratio 5.9), students who smoke cigarettes compared to those who do not (*p* = 0.008, odds ratio 10.7), students who smoke shisha compared to those who do not (*p* = 0.001, odds ratio 17.8), and students who have friends who smoke e-cigarettes compared to those who do not (*p* = 0.013, odds ratio 8.7) were found to be significant predictors for e-cigarette smoking.

## Discussion

The current study revealed that 7.3% of the participating medical students used e-cigarettes. In similar studies conducted in Pakistan [22], Turkey [23] Qassim [24], and Makkah [25], the prevalence of e-smoking among medical students was 6.3%,11.5%, 10.6%, and 31.8% respectively while in a US survey among medical students, only 4.1% of them reported having ever tried e-cigarettes [26]. There was a statistically significant higher percentage of e-cigarette use among students in the clinical years (11.3%) compared to those in the preclinical years (3.3%) with a *p*-value of 0.008. The majority of e-cigarette users were males. This finding is similar to other studies from Pakistan [22], Turkey [23], and the USA [27].

The results of this study demonstrated that about half of the students who used e-cigarettes smoked traditional cigarettes (and shisha) as well. A similar study in Turkey also reported that a higher percentage of e-cigarette use was found among active smokers [24].

A fairly high percentage of medical students in the present study were aware of e-cigarettes. In a study conducted in Turkey, students indicated that friends were the main source of knowledge about e-cigarettes [22]. In contrast, the main source of e-cigarette awareness in the current study and a similar study conducted in Jeddah, SA was social media [28]. The next source of the participants’ e-cigarette knowledge in the current study was their friends. In addition, having friends who use e-cigarettes was a significant predictor of e-smoking. Similarly, in a Saudi study, more than two-thirds of e-cigarette smokers reported having an e-cigarette user friend and admitted the friend’s role in encouraging them to try e-cigarettes [29]. This emphasizes the effect of peers on an individual’s behavior and attitude. This finding highlights the importance of incorporating education about e-cigarettes into the medical school curriculum. This will equip future physicians with adequate knowledge and expertise, enabling them to discuss the issue of e-smoking as well as the other forms of nicotine products with their patients.

A higher percentage of e-cigarette smokers, current smokers, and ex-smokers in the present study reported that e-cigarettes are less harmful than traditional cigarettes. The same finding was reported by a study conducted in Saudi Arabia [28], and in Turkey [22]. Similarly about one-fifth of the medical students at Umm Al Qura University, SA reported that e-cigarettes are less harmful than traditional cigarettes [25].

Similarly, a higher percentage of current smokers and e-cigarette smokers, and students in the clinical years reported that e-cigarettes are not addictive compared to their non-smoker counterparts or their younger colleagues in the preclinical years. This is in contrast to a US study where more of the older medical students reported that e-cigarettes were addictive compared to their younger counterparts. The difference between the two studies may be attributed to the educational lecture about e-cigarettes that the US students received during the study [27].

A study performed in the USA demonstrated a significant association between the intensive use of e-cigarettes and smoking cessation[30]. Interestingly, the majority of current smokers and ex-smokers in the current study believed that e-cigarettes help in smoking cessation. About a quarter of participants at Umm Al-Qura University and Qassim University, SA thought that e-cigarettes help in smoking cessation [25]. Similarly, in the current study, 24.3% of the participants considered that e-cigarettes are helpful in smoking cessation [16]. In contrast, Jeddah’s study found that 42.7% of e-cigarette users reported that they used it as a smoking cessation aid, and about half of them succeeded [25].

In the present study, a greater proportion of ex-smokers (66.7%) and current smokers (56.8%) indicated that they would recommend e-cigarettes to other smokers. This finding highlights the significant influence of peer dynamics on vaping behaviors among youth. A similar finding was noted by a meta-analysis that emphasized that peer smoking behaviors significantly influence youth decisions to try e-cigarettes [31].

Although e-cigarette use may pose risks to both maternal and child health [30], a higher percentage of e-cigarette users in the present study believed that e-cigarettes are not harmful to pregnant women compared to non-e-cigarette users. Additionally, about one-third of the participants reported that they did not know. A similar finding was reported by a study from Pakistan, where the majority of e-cigarette users thought that it was not harmful during pregnancy [22].

### Study limitations

The study was limited to Cairo University medical students, which limits the generalizability of the results to the general population of Egypt.

Further research including medical students from different universities and graduated physicians is recommended. In addition, an in-depth assessment of physicians' knowledge and experience with e‑cigarettes is needed in order to bridge the detected knowledge gap.

## Conclusion

This study revealed a gap in medical students’ knowledge regarding electronic cigarettes, which reflects a gap in medical education regarding this issue. With the increasing prevalence of e-cigarette use, medical students must receive more education about such a public health concern. More research and education are essential to address the safety of using e-cigarettes as well as their efficacy as a smoking cessation tool. Empowering medical students and medical school graduates with correct information through curricular and extracurricular activities will enable them to confidently discuss e-cigarette use with their patients and offer them the appropriate evidence-based advice.

## Data Availability

The datasets used and/or analyzed during the current study are available from the corresponding author upon reasonable request.

## Abbreviations

E-cigarettes: Electronic cigarettes
FDA: Food and Drug Administration
WHO: World Health Organization
